# Modelling the force of infection for hepatitis B and hepatitis C in injecting drug users in England and Wales

**DOI:** 10.1186/1471-2334-6-93

**Published:** 2006-06-08

**Authors:** AJ Sutton, NJ Gay, WJ Edmunds, VD Hope, ON Gill, M Hickman

**Affiliations:** 1 Centre for Research on Drugs and Health Behaviour, Department of Primary Care and Social Medicine, Imperial College London, London W6 8RP, UK; 2 Health Protection Agency, Centre for Infections, 61 Colindale Ave, London NW9 5EQ, UK

## Abstract

**Background:**

Injecting drug use is a key risk factor, for several infections of public health importance, especially hepatitis B (HBV) and hepatitis C (HCV). In England and Wales, where less than 1% of the population are likely to be injecting drug users (IDUs), approximately 38% of laboratory reports of HBV, and 95% of HCV reports are attributed to injecting drug use.

**Methods:**

Voluntary unlinked anonymous surveys have been performed on IDUs in contact with specialist agencies throughout England and Wales. Since 1990 more than 20,000 saliva samples from current IDUs have been tested for markers of infection for HBV, HCV testing has been included since 1998. The analysis here considers those IDUs tested for HBV and HCV (n = 5,682) from 1998–2003. This study derives maximum likelihood estimates of the force of infection (the rate at which susceptible IDUs acquire infection) for HBV and HCV in the IDU population and their trends over time and injecting career length. The presence of individual heterogeneity of risk behaviour and background HBV prevalence due to routes of transmission other than injecting are also considered.

**Results:**

For both HBV and HCV, IDUs are at greatest risk from infection in their first year of injecting (Forces of infection in new initiates 1999–2003: HBV = 0.1076 95% C.I: 0.0840–0.1327 HCV = 0.1608 95% C.I: 0.1314–0.1942) compared to experienced IDUs (Force of infection in experienced IDUs 1999–2003: HBV = 0.0353 95% C.I: 0.0198–0.0596, HCV = 0.0526 95% C.I: 0.0310–0.0863) although independently of this there is evidence of heterogeneity of risk behaviour with a small number of IDUs at increased risk of infection. No trends in the FOI over time were detected. There was only limited evidence of background HBV infection due to factors other than injecting.

**Conclusion:**

The models highlight the need to increase interventions that target new initiates to injecting to reduce the transmission of blood-borne viruses. Although from the evidence here, identification of those individuals that engage in heightened at-risk behaviour may also help in planning effective interventions. The data and methods described here may provide a baseline for monitoring the success of public health interventions.

## Background

Injecting drug use is a key risk factor, and injecting drug users (IDUs) are a core group for several infections of public health importance, especially hepatitis B (HBV) and hepatitis C (HCV). In England and Wales, where less than 1% of the population are likely to be IDUs [1], approximately 38% of laboratory reports of HBV [2], and 95% of HCV reports[2] are attributed to injecting drug use.

A key measure of transmission within a given population is the force of infection (FOI). This is defined as the instantaneous per capita rate at which susceptibles acquire infection and reflects the degree of contact with potential for transmission between susceptibles and infecteds[3]. The aim of this study is to estimate FOI for HBV and HCV in the IDU population in England and Wales and how this may have evolved, both over time and as IDUs' injecting careers progress.

Analysis that includes only a single infection can estimate the mean FOI but not the variance. A model is proposed here that considers HBV and HCV simultaneously and fits to observed data on the prevalence of these infections from a survey of IDUs with markers of single and multiple infections. The effects of individual heterogeneity within the IDU population are investigated, while the proposed model also considers the transmission of HBV infection in IDUs from non-IDUs.

## Methods

### Data

Since 1990, voluntary unlinked anonymous (UA) oral fluid samples have been collected from injecting drug users in contact with specialist agencies throughout England and Wales [4,5]. These agencies provide services including needle exchange, methadone maintenance and outreach work. Behavioral information is collected through a brief anonymous questionnaire unlinked from client identifying information. The fields used in this analysis were: Year Surveyed, age at first injection, age when surveyed, injected in the last 4 weeks, ever vaccinated against HBV, and the number of doses of HBV vaccine received.

Oral fluid samples were tested for antibody to HBV (anti-HBc) and from 1998; HCV. The sensitivity and specificity of the test for anti-HBc was 75% and 100% respectively, and 83% and 100% respectively for anti-HCV [6].

**Figure 1:**
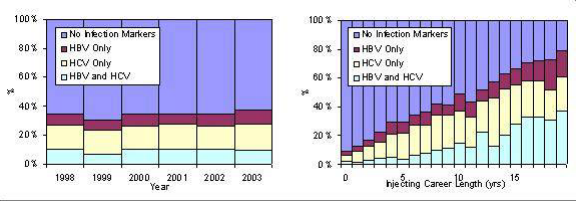
The percentage of the surveyed IDU population by infection status over injecting career length and time.

IDUs were included in this analysis if they reported having injected in the four weeks prior to being surveyed. Samples from 1990 to 1997 were not tested for HCV and so were excluded leaving six complete consecutive surveys 1998–2003 containing 12,826 records of current IDUs. Only IDUs with an unequivocal result for both tests were included in this analysis (leaving 12,814 records). The data were further constrained by limiting the current age range at the time of the survey to between 16–49 years, and the age of first injection to be from 13–45 years (12,031 records). As the FOI considers the rate that susceptibles acquire infection only those persons that were unvaccinated against HBV were considered in this analysis. (Unvaccinated IDUs were defined as those that answer no to the question of having been vaccinated against HBV and report having received no doses of HBV vaccine) (6,269 records). The percentage of the population with anti-HBc, anti-HCV, and dual infection with variation in injecting career length and over time is shown in Figure 1. An injecting career length of each IDU was calculated from this data by considering the difference between the current age and the age of first injecting. Due to paucity of data, those IDUs with an injecting career length of 20 years or more were omitted from this analysis, leaving 5,682 reports from IDUs to be considered here. The impact of changing this cut-off was considered during sensitivity analysis.

#### Model

The status of each IDU is considered with respect to both infections. To introduce individual heterogeneity of at-risk behaviour into the model we introduce a frailty Z which represents an individual's relative rate of infection. An individual of frailty Z and career length *τ *at time t has the risk  of previous HBV infection and  of previous HCV infection. The frailty distribution is assumed gamma with shape *θ *and scale parameter = 1.

For infection acquired through injecting drug use only, let *π*_00_(*τ*, *t*), *π*_*B*0_(*τ*, *t*), *π*_0*C*_(*τ*, *t*) and *π*_*BC*_(*τ*, *t*) denote the proportion of IDUs in year t with an injecting career length of *τ *that are; uninfected, infected by HBV not HCV, infected by HCV not HBV, and both HBV and HCV respectively. Then







*π*_*BC*_(*τ*, *t*) = 1 - *π*_00_(*τ*, *t*) - *π*_*B*0_(*τ*, *t*) - *π*_0*C*_(*τ*, *t*)

where:

Λ_*B*_(*τ*, *t*) and Λ_*C*_(*τ*, *t*) are the cumulative force of infection for HBV and HCV respectively in year t for injecting career length *τ*.

These equations represent a reparameterisation of Farrington's [7] formulation so that the total prevalence of each infection is independent of *θ *(see Appendix 2).

#### HBV background prevalence and test sensitivity

Background HBV prevalence is included in the model and reflects the possibility of transmission of HBV from outside the IDU population. As 95% of reports with exposure data to HCV indicate injecting drug use [2], it has been assumed here that there was no non-injecting related transmission of HCV. To incorporate HBV background prevalence and the sensitivity of the HBV and HCV tests into this model the equations describing the prevalence of the two viruses *π*_*xy*_(*τ*, *t*)have been modified to both reflect the possibility that HBV infection can occur for reasons other than injecting and that the tests for HBV and HCV have a sensitivity and specificity that is less than 100%. It is assumed in this model that the risk of background HBV infection is constant through time and injecting career length, and that there is no possibility of background infection from HCV.

*P*_0*y *_= *π*_0*y*_(1 - *b*)

*P*_*By *_= *π*_*By *_+ *π*_0*y*_*b*

and

*V*_00 _= *P*_00 _+ (1 - *s*_*B*_)*P*_*B*0 _+ (1 - *s*_*C*_)*P*_0*C*_+ (1 - *s*_*B*_) (1 - *s*_*C*_)*P*_*BC*_

*V*_*B*0 _= *P*_*B*0_*s*_*B *_+ *s*_*B*_(1 - *s*_*C*_)*P*_*BC*_

*V*_0*C *_= *P*_0*C*_*s*_*C *_+ *s*_*C*_(1 - *s*_*B*_)*P*_*BC*_

*V*_*BC *_= *s*_*B*_*s*_*C *_*P*_*BC*_

where

b = background prevalence of infection of HBV.

s_B _= sensitivity of the HBV test

s_C _= sensitivity of the HCV test

P_xy _= proportion of IDUs in year t with an injecting career length of *τ *with status xy allowing for the background prevalence of infection of HBV.

V_xy _= proportion of IDUs in year t with an injecting career length of *τ *who have test status xy (allowing for the sensitivity of the HBV and HCV tests)

#### Parameterisation

The cumulative FOI in 1998 (the first year of data considered here for each virus) by injecting career length, Λ(1998, *τ*) is described by the function *f*(*τ*). This is described by a four parameter logistic function.



The cumulative FOI in 1998 describes all infection in previous years and is estimated with the FOI for the more recent years (1999–2003). No attempt has been made to reduce the parameters describing this function with the priority being to ensure sufficient flexibility and a good fit to the data.

The force of infection from 1999 onwards is modelled as the product of a function describing its trend over time g(*t*) and a function describing its trend with injecting career length *h*(*τ*) [8]:

*λ *(*t*, *τ*) = *g*(*t*)*h*(*τ*)

To standardize results for each virus, *h*(0) is fixed equal to 1. Both *g*(*t*) and *h*(*τ*) are parameterized on piecewise constant functions

The initial model used at the start of the analysis is defined as describing function *f*(*τ*) for each virus with a four parameter logistic function (8 parameters), and functions *g*(*t*) and *h*(*τ*) for each virus are described by an individual value for each year 1999–2003 (10 parameters) and injecting career length 1–19 years respectively (38 parameters). Along with the parameters; frailty *θ *and HBV background prevalence *b*, this leads to the initial model being described by 58 parameters.

It is assumed that IDUs who report an injecting career length of 0 years have been injecting for an average of 6 months (see Appendix). IDUs who appear in the UA surveys such as those considered here, are recruited from those in contact with services. The probability of being in contact with services increases with injecting career length[9] and therefore the average career length of surveyed new initiates may be higher than the 6 months assumed. Because the estimated FOI in new initiates will be correlated with the duration of exposure (injecting career length) we investigate the sensitivity of our estimates to the career length assumed. As an extreme case, it is assumed that IDUs with a reported injecting career length of 0 years have been injecting for 12 months, while applying the injecting career length of the remaining IDUs at reported levels.

### Model fitting

If *n*_*xy*_(*τ*, *t*) denotes the number of individuals in year t with injecting career length *τ *with test results coded *xy *as above, the log-likelihood (L) is the product multinomial.



Beginning with an initial model and then maintaining the four parameter logistic function for *f*(*τ*), backwards-stepwise elimination was used to reduce the number of parameters describing *g*(*t*) and *h*(*τ*). Models were compared using the analysis of deviance with the Chi-squared test, the criteria for dropping parameters being that p > 0.05. When the parsimonious model (the best fitting model with the fewest parameters) had been identified, confidence intervals were calculated using profile likelihood. For both functions *g*(*t*) and *h*(*τ*) a range of alternative reduced models were considered including alternative values for the fixed category of *h*(0) e.g. 0 years 0–1 years, 0–2 years etc. A Selection of the reduced models examined during the backwards-stepwise elimination process is described in the following (only changes from the initial model are noted):

1. Initial model

2 *h*(*τ*) for each virus is grouped into 4 injecting career length groups (1–2 yrs, 3–4 yrs, 5–9 yrs, 10+yrs, 0 yrs is fixed)

3 As model 2 above, except *h*(*τ*) is the same for each virus.

4 As model 3 above, except *h*(*τ*) is described by 1 injecting career length group (1+ years, 0 yrs is fixed)

5 As model 4 above, except *g*(*t*) for each virus is a single parameter (1999–2003).

6 As model 5 above, except function *h*(*τ*) = 1.

## Results

The calculated model parameters for the parsimonious model are shown in Table 1 with 95% confidence intervals. The results from the model suggest that HBV background prevalence is low (= 0.00, 95% C.I: 0–0.0161).

**Table 1:** Parameter values describing the parsimonious model

**Model Parameters**	**HBV**	**95% C. I.**	**HCV**	**95% C. I.**	**Global**	**95% C. I.**
*g*(*t*) trend in the FOI by year 1999–2003						
1999–2003	0.1079	(0.0840–0.1327)	0.1608	(0.1314–0.1942)		
*h*(*τ*) trend in the FOI by injecting career length						
<1	1	fixed	Same as HBV			
1+	0.3272	(0.2359–0.4443)	Same as HBV			
HBV background prevalence *b*					0	(0.0–0.0161)
Frailty *θ*					0.6566	(0.5305–0.8224)
	U -0.132		-2.524			
	V 0.117		0.026			
	W 63.99		77.52			
	Z 295.5		19.49			

Table 2 shows a summary of the results of the fitting procedure. The initial model gave a good fit to the data and successive reducing in the number of parameters in models 2–5 did not significantly worsen the fit. Model 6 considered the impact of removing the function *h*(*τ*), however this provided a significantly less good fit than Model 5 (p < 0.001). Model 5, therefore, was taken to be the parsimonious model.

**Table 2:** Goodness of fit for initial and reduced models

**Model**	**d.f. (n = 471)**	**Deviance**	**p value**
1 (Initial Model)	413	392.2	
2	443	413.6	0.87
3	447	415.6	0.73
4	450	417.9	0.52
5	458	428.8	0.21
6	459	469.6	<0.0001

**Figure 2:**
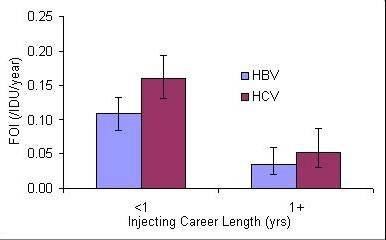
The force of infection for HBV and HCV with variation in injecting career length for 1999–2003.

**Figure 3:**
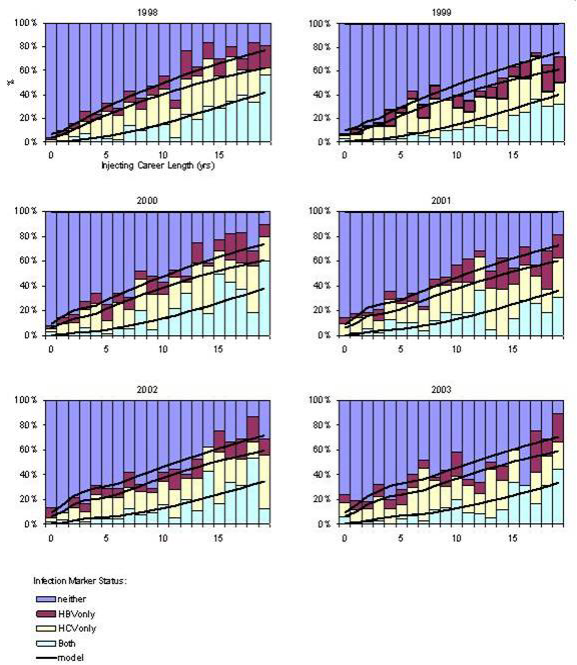
Model fit to data for each survey 1998–2003.

Figure 2 shows the estimated FOI by injecting career length for HBV and HCV. The parameter values for the parsimonious model are shown in Table 2, while the model fit to data for each survey is shown in Figure 3. Across similar injecting career lengths the FOI was found to be higher for HCV than HBV. For both HBV and HCV the FOI is higher for new initiates to injecting (injecting career length <1 year) (Figure 2) compared to experienced IDUs.

**Figure 4:**
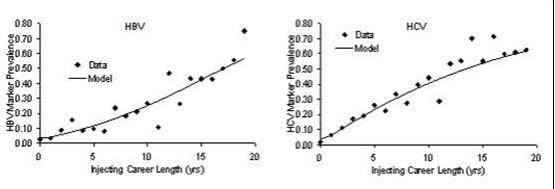
The model to data fit for 1998 survey data describing the prevalence of HBV and HCV infection.

The model fit to data for HCV and HBV in 1998 is shown in Figure 4. The close fit of the model to the data provides additional confidence in the selection of the four parameter logistic function to describe function *f*(*τ*).

**Figure 5:**
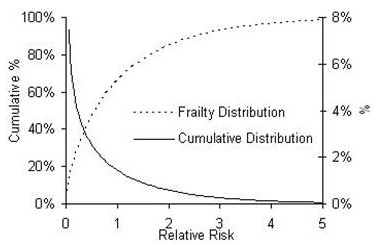
The estimated frailty distribution and a cumulative density function describing the frailty for HBV and HCV.

The estimated frailty distribution for the IDU population is shown in Figure 5. It can be seen that there is strong evidence of individual heterogeneity, with the majority of IDUs (64%) having a FOI less than the average (relative risk<1), and a small proportion (15%) having a FOI much higher than average (relative risk >2).

To investigate the impact of excluding those IDUs with an injecting career length of 20 years or greater, a further reduction was made with IDUs with an injecting career length of greater than 10 years being excluded from the analysis. However model results were found to be similar (not shown) thereby suggesting no good reason to further reduce the number of IDUs included in this analysis.

The importance of considering the test sensitivity for HBV and HCV in the model was shown when these are excluded from the model. The resultant force of infection estimates for both HBV and HCV were found to be lower than when test sensitivity is included in the model (results not shown). It has been assumed that all new initiates to injecting (injecting career length = 0 yrs) have been injecting for an average six months. However if instead it is assumed that all IDUs in the surveys have been injecting for at least 1 year, then this lessens (but does not remove) some of the injecting career length effect on the FOI (*h*(1+) = 0.5704, 95% C.I: 0.4471–0.7801) although as before the strong evidence of individual heterogeneity remains (*θ *= 0.6547, 95% C.I: 0.5301–0.8208).

## Discussion

Unlinked Anonymous data collected from IDUs in England and Wales was analysed to estimate the trend in the force of infection (FOI) for HBV and HCV in IDUs over time and career length. By considering both viruses together we assessed the heterogeneity of risk within the IDU population and the effect of background HBV transmission due to transmission between IDUs and non-IDUs.

The IDUs recruited into the survey are self-selected and in contact with specialist agencies and may not be representative of the whole IDU population. It has been shown that HCV prevalence tends to be higher among IDU recruited from treatment sites, though generally after adjustment for age and injecting career any difference is substantially reduced[10]. It has been established that the time between onset and presentation to treatment varies, and that some IDU will never enter treatment[11]. Thus, an important potential bias arises if injectors in treatment and sampled by the survey are different with respect to occurrence of HCV and HBV[12] when compared to the overall IDU population. For example if there is a greater difference in HCV prevalence among new initiates contacting and not contacting services than among experienced IDUs, then this will influence the estimated difference in FOI between new and experienced IDUs compared to the actual FOI in the overall IDU population. It has been assumed that the risk behaviour in the surveyed IDU population considered here is the same as the IDU population in England and Wales and additionally there is the same likelihood of a positive or negative IDU being surveyed as there would be in the IDU population in England and Wales.

The model suggest that the FOI of HBV and HCV is up to four times higher among new initiates (injecting career length <1 year) than for IDUs with longer injecting careers, a finding which is supported by previous studies[13,14]. In all cases it was found that there is increased risk of infection in new initiates compared to experienced IDUs. The scale of these findings must be approached with caution as they are sensitive and dependent on information about and from a small sub-group of IDUs (those with injecting career lengths of less than 1 year). It has been assumed that IDUs reporting an injecting career length of 0 years (i.e. start age = current age), have an even chance of their exact injecting career length being anything from 0 days to 1 year. However this does not allow for the delay from the initiation of injecting until coming into contact with services. If it is assumed instead that all current IDUs (including new initiates) in the surveys have been injecting for at least one year, the injecting career length effects are lessened with new initiates only having FOI estimates for HBV and HCV up to twice as high as experienced IDUs.

The model indicates considerable heterogeneity in the FOI among the sample suggesting some IDUs are at significantly greater risk from infection from blood-borne viruses within the larger IDU population. Analysis that considers only a single infection cannot address the issue of individual heterogeneity, or its effect on other estimates. In such analysis the presence of high risk individuals within the IDU population would cause an apparently higher FOI in IDUs with short injecting career lengths. We find such a career length effect after allowing for individual heterogeneity. These results demonstrate the added value of this combined analysis. From this data alone it is impossible to draw any conclusions about the reasons for this individual heterogeneity, although it could be due to certain groups within the overall IDU population stratified by ethnicity, socioeconomic status, sex, or other demographic variables.

It is acknowledged that the prevalence of each virus within the IDU population may be due to infection from outside the IDU population particularly for HBV. However the low prevalence of background HBV infection suggests that the infection in the IDU population is primarily due to contact between IDUs (needle sharing or sexual contact).

During this analysis we excluded those IDUs that reported being vaccinated against HBV. This was done as their inclusion would result in an underestimation in the FOI estimates obtained for HBV. When individuals are diagnosed with HCV infection, due to complications associated with dual infection they should be vaccinated against HBV [15]. A consequence of this is that some individuals that are HCV positive and vaccinated against HBV were excluded from this analysis. As the prevalence of HCV is higher in those IDUs that are excluded from this analysis (vaccinated against HBV) compared to those included (RR = 1.15, 95% C.I: 1.09–1.21 adjusting for injecting career length), the results here may underestimate the FOI in IDUs for HCV. To overcome this and to allow a greater number of IDUs to be included in the analysis, future models should incorporate an HBV vaccination rate that varies based on an IDU's HCV status, this will lead to a greater number of IDUs being included in this analysis (vaccinated and unvaccinated) and may help to remove any bias in the FOI estimates obtained here. An additional advantage of this is that an increase in the number of HCV positive individuals included in this analysis may also increase power to detect any changes in the FOI over time.

Future work could consider whether the results about individual heterogeneity in terms of frailty could be compared to data describing the heterogeneity of risk behaviour within the IDU population through injecting or sharing behaviour. This would help to corroborate the findings obtained here.

## Conclusion

The estimation of the FOI from serial prevalence data provides added epidemiological value. Previous authors have studied the incidence of infection in a cohort of IDUs [16] although to the authors' knowledge no previous studies have used this method of modelling to estimate the FOI for HBV and HCV in the IDU population. The models highlight the need to increase interventions that target new initiates to injecting to reduce the transmission of blood-borne viruses. Although from the evidence here, identification of those individuals that engage in heightened at-risk behaviour should be undertaken. The data and methods described here provide a baseline for monitoring the success of public health interventions.

## Competing interests

The author(s) declare that they have no competing interests.

## Authors' contributions

AJS contributed to designing and planning the study, created the force of infection model and carried out all analysis, interpreted the results and prepared the manuscript as the lead writer. NJG helped develop the model and interpret the data and results. WJE, VDH, ONG, and MH contributed to the study development and helped in the interpretation of the results. All authors read and provided comments on the manuscript and approved the final paper.

## Appendix

### Force of infection

The FOI (*λ*(*τ*,t)), the rate at which susceptible IDUs acquire infection [17], may vary with time (*t*) and injecting career length (*τ*). The prevalence *P*(*τ*, *t*) quantifies the expected proportion of individuals with injecting career length *τ *who were antibody positive at time *t *[18].

Prevalence in year t for those who have injected for *τ *years is:

*P*(*τ*, *t*) = 1 - *e*^-Λ(*τ*, *t*)^

where Λ(*τ*, *t*) is the cumulative force of infection in year t for those who have injected for *τ *years and is given by:



this may be expressed relative to a baseline year T



Where *τ*_0_= max(0, *τ *- (*t *- *T*)) is the career length at time T.

The cumulative FOI for each reported injecting career length is calculated by averaging over the range of possible career lengths. As previously discussed the reported injecting career length is calculated by considering the difference between an IDU's age at first injection and current age when surveyed.

Therefore an IDU with a reported injecting career length = A years, may have been injecting from (A-1) years + 1 day to (A+ 1) years - 1 day.

For those IDUs that have been injecting from (A-1) years + 1 day to A years.

The average cumulative  where Λ_0_= cumulative FOI up to A-1 years and *λ*_1_is the FOI from A-1 to A years.

For those IDUs that have been injecting from A years + 1 day to A+1 years - 1 day the average cumulative  where *λ*_2_is the FOI from A to A-1 years. Therefore the average cumulative FOI experienced by IDUs reporting an injecting career length of A is



## Appendix 2

Beginning with the following equations proposed by Farrington et al (2001) [7].







*π*_*BC*_(*τ*, *t*) = 1 - *π*_*B*0_(*τ*, *t*) - *π*_0*C*_(*τ*, *t*) - *π*_00_(*τ*, *t*)

The variables Y_B _and Y_C _should not be interpreted as the cumulative FOI for HBV and HCV respectively because the total prevalence of each infection depends on *θ*. We re-parameterize these equations so that the total prevalence of each infection is independent of *θ *as shown below.

We define



and



These are then substituted into Farrington's equations above giving:







*π*_*BC*_(*τ*, *t*) = 1 - *π*_00_(*τ*, *t*) - *π*_*B*0_(*τ*, *t*) - *π*_0*C*_(*τ*, *t*)

where:

Total prevalence of HBV = *π*_*B*0 _+ *π*_*BC*_= 

Total prevalence of HCV = *π*_*C*0 _+ *π*_*BC*_= 

And therefore:

Λ_*B*_(*τ*, *t*) and Λ_*C*_(*τ*, *t*) are the cumulative force of infection for HBV and HCV respectively in year t for injecting career length *τ*.

## Pre-publication history

The pre-publication history for this paper can be accessed here:


